# Critical Review of Glyphosate-Induced Oxidative and Hormonal Testicular Disruption

**DOI:** 10.3390/antiox14091036

**Published:** 2025-08-22

**Authors:** Sara R. Branco, Marco G. Alves, Pedro Fontes Oliveira, Ariane Zamoner

**Affiliations:** 1 LAQV-REQUIMTE and Department of Chemistry, University of Aveiro, 3810-193 Aveiro, Portugal; sarabranco@ua.pt; 2 Department of Medical Sciences, Institute of Biomedicine (iBiMED), University of Aveiro, 3810-193 Aveiro, Portugal; marcoalves@ua.pt; 3 Laboratory of Biochemistry and Cell Signaling—LaBioSignal, Department of Biochemistry, Center of Biological Sciences, Federal University of Santa Catarina, Florianópolis 88037-000, SC, Brazil

**Keywords:** glyphosate, Sertoli cells, Leydig cells, male fertility, metabolism, endocrine disruption, AMPA, antioxidants

## Abstract

Glyphosate, the active ingredient in many herbicides, has been extensively used in agricultural practices worldwide, leading to environmental persistence of the herbicide and its main metabolite, aminomethylphosphonic acid (AMPA), particularly in water and soil. Despite a short half-life in biological fluids, frequent detection of glyphosate and AMPA in urine samples suggests ongoing human exposure. Evidence indicates that glyphosate and AMPA may exert endocrine-disrupting effects on testicular function. Glyphosate exposure may disrupt the hypothalamic–pituitary–gonadal axis, impacting serum testosterone levels and other key hormones involved in spermatogenesis and fertility. It has also been shown to impair key cellular processes within the male reproductive system, including oxidative stress, mitochondrial dysfunction, and hormone biosynthesis. These findings raise concerns about the herbicide’s ability to compromise sperm production, structure, and motility, which are crucial factors for male fertility. This review examines the mechanisms underlying glyphosate-induced testicular toxicity, emphasizing endocrine disruption, oxidative stress, and mitochondrial dysfunction, and highlights the need for further studies on long-term effects across different life stages and genetic backgrounds. Glyphosate-induced testicular toxicity can be counteracted by antioxidant agents, which emerge as promising therapeutic strategies in need of further investigation.

## 1. Introduction

Over recent decades, a decline in human fertility rates has been observed, with infertility becoming a significant global health issue. According to the World Health Organization (WHO), infertility is the inability to conceive after at least 12 months of regular, unprotected sexual intercourse. This condition is considered a major health problem and is estimated to affect 7–15% of couples worldwide, with approximately 50% related to a male factor [1]. Several factors can contribute to the rising trend of male infertility, which can be stratified as acquired, congenital, and idiopathic. Among idiopathic risk factors, environmental or occupational exposure to contaminants seems to play a significant role. Indeed, numerous studies have demonstrated that these compounds can adversely affect the male reproductive system, with reported outcomes including alterations in testicular structure [2], the induction of apoptosis in testicular cells [3], and disruption of spermatogenesis [4]. Glyphosate is a biocide with a broad-spectrum activity, and it is currently the most widely used herbicide worldwide. Its extensive use has led to widespread environmental distribution, resulting in its accumulation in food products, soil, and water [5]. Consequently, human populations experience continuous exposure to glyphosate and its primary metabolite, aminomethylphosphonic acid (AMPA), raising concerns about potential adverse health outcomes. Although glyphosate remains a staple for weed management, its potential reproductive toxicity and environmental impact are subjects of ongoing and significant concern. This review critically examines the current body of knowledge regarding the effects of glyphosate exposure on male reproductive health, encompassing a comprehensive analysis of available studies, focusing on impacts to spermatogenesis, hormone regulation, and testicular morphology. In this context, oxidative stress has emerged as a key unifying mechanism linking glyphosate exposure to male reproductive toxicity, including impaired spermatogenesis, mitochondrial dysfunction, and altered antioxidant defenses.

## 2. Methodology

A critical and comprehensive literature review was carried out using the PubMed database, encompassing publications available up to December 2024. The search focused on studies investigating the effects of glyphosate and glyphosate-based herbicides (GBHs) on male reproductive health. Keywords and Boolean combinations used in the search strategy included: “glyphosate,” “glyphosate-based herbicides,” “male fertility,” “spermatogenesis,” “testicular toxicity,” “testosterone,” “estradiol,” “Leydig cells,” “Sertoli cells,” “oxidative stress,” oxidative damage,” “redox system,” “mitochondrial dysfunction,” “antioxidants,” “testis,” “endocrine disruption,” “mitochondrial dysfunction,” and “reproductive hormones.” The inclusion criteria comprised original experimental studies conducted in vitro and in vivo, as well as observational studies and mechanistic investigations in both animal models and humans. Scientific rigor was evaluated based on experimental design, sample size, appropriate statistical methodology, and the presence of control groups. Studies that did not meet minimum standards of experimental control or methodological clarity were excluded from the review. There was no exclusion of articles based on language; when necessary, articles published in languages other than English were translated using Google Translate to facilitate their inclusion and interpretation. Studies were selected for this review based on their scientific rigor and relevance to the scope. When reported, both the administered dose (mg/kg/day) and the concentration in the exposure medium (e.g., mg/L in drinking water or diet) were recorded. In studies where only one of these metrics was available, the other could not be derived unless fluid or food intake data were provided in the original publication. Additionally, reference lists of relevant publications were manually screened to identify further eligible studies and ensure comprehensive coverage of the topic. Selected studies were critically evaluated with emphasis on mechanistic insights, cellular and molecular targets, and implications for testicular male fertility.

## 3. Historical Context and Emerging Concerns in Glyphosate Usage

Glyphosate (IUPAC name: N-(phosphonomethyl) glycine) is an herbicidal glycine amino acid derivative first synthesized in 1950 by the Swiss chemist, Dr. Henri Martin. Due to the absence of pharmaceutical properties, the molecule was sold to other companies, which led to various trials aimed at unraveling the potential applications. It was not until the early 1970s that its agricultural potential came to light when Dr. John Franz, an organic chemist at Monsanto Company, recognized glyphosate’s herbicidal capacities. This discovery facilitated the incorporation of glyphosate as the primary component in glyphosate-based herbicides (GBHs), namely Roundup^®^, a Bayer’s commercial formulation originally developed by Monsanto, the best-known registered trademark example [6]. Chemically, glyphosate belongs to a group of compounds known as phosphonates, more precisely a phosphonic acid resulting from the formal oxidative coupling of methyl phosphonic acid with a glycine amino group [7]. This group of molecules is characterized by the presence of an unusual carbon-phosphorus (C-P) bond, which provides unique chemical and physical properties, such as high adsorption, high solubility in water, and high compatibility with other chemical substances [8]. It is a non-volatile substance, stable in air and resistant to photochemical degradation [9]. Formulations of glyphosate include ammonium, diammonium, dimethylammonium, isopropylamine, and potassium salts, which enhance its solubility while preserving its efficacy as an active ingredient [7]. Two decades after the discovery of its herbicidal properties, the introduction of glyphosate-resistant crops, primarily soybeans, maize, and cotton varieties, revolutionized its usage as a broad-spectrum, post-emergent herbicide. This discovery dramatically extended the period during which GBHs could be applied [6]. Since then, glyphosate has gained global recognition, approved for controlling over 150 weed species in silviculture, domestic, and urban areas in 130 countries [10]. The expansion of glyphosate applications has led to a significant increase in its usage [11], making i the most extensively used herbicide, reaching an estimated annual volume of 700,000 tons [12].

### 3.1. Herbicidal Mechanism of Action and Metabolism of Glyphosate

**Figure 1:**
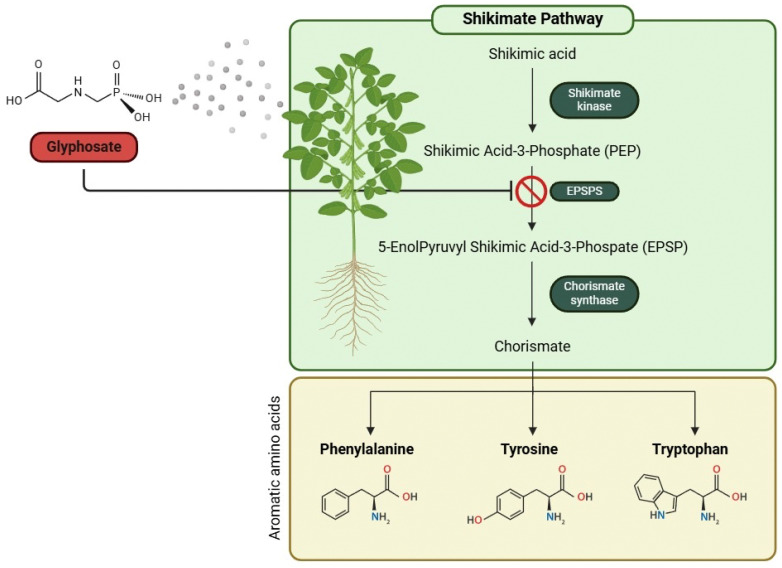
Glyphosate’s mode of action. Glyphosate inhibits the enzyme 5-enolpyruvylshikimate-3-phosphate synthase (EPSPS) in the shikimate pathway, crucial for the biosynthesis of aromatic amino acids (phenylalanine, tyrosine, and tryptophan), which are essential for protein synthesis and the production of various plant metabolites. Consequently, EPSPS inhibition disrupts these processes, resulting in the death of the targeted organism.

Glyphosate is typically applied to plants’ foliage, being absorbed through the leaves, the trunk, or the roots. Upon entry into the plant, it quickly migrates to regions of active growth [9]. The mechanism of glyphosate herbicidal action is associated with its ability to inhibit the shikimate pathway. This pathway includes seven enzymatic reactions, beginning with the condensation of phosphoenolpyruvate (PEP) and erythrose 4-phosphate (E4P), culminating in the synthesis of chorismate, the central precursor for all aromatic amino acids and several metabolites in plants [13,14]. Glyphosate inhibits the enzyme 5-enolpyruvylshikimate-3-phosphate synthase (EPSPS), the sixth enzyme on the shikimic acid pathway. This enzyme catalyzes the transfer of the enolpyruvyl moiety of PEP to shikimate-3-phosphate (S3P), resulting in 5-enolpyruvylshikimate-3-phosphate (EPSP) and inorganic phosphate. Glyphosate competes with PEP, inhibiting EPSPS by occupying the PEP binding site of the S3P complex. EPSPS is crucial for the synthesis of aromatic amino acids phenylalanine, tyrosine, and tryptophan, which are essential building blocks of proteins and are also precursors to several plant metabolites [15]. The inhibition of this pathway leads to a reduction in the synthesis of aromatic amino acids (Figure 1), subsequently affecting protein synthesis, ultimately leading to the targeted organism’s death, within a few days [16,17].

In addition to its widespread application as an herbicide for weed control, glyphosate could also be used as a systemic desiccant in pre-harvest processes for various crops. Unlike traditional desiccants, glyphosate’s systemic action causes gradual plant desiccation, taking weeks rather than days for complete die-back and drying. This extended action may lead to its persistence not only in the soil but also in food products, potentially increasing exposure risks for consumers and impacting environmental health. Once released in the environment, glyphosate undergoes a gradual degradation in dead plant material, soil, and water, facilitated by various microorganisms [18]. It undergoes metabolic degradation via two major pathways, leading to the production of the metabolites AMPA, the keystone metabolite of glyphosate, and sarcosine [19]. In the pathway leading to AMPA production, glyphosate’s carboxyl group is initially cleaved by the enzyme glyphosate oxidoreductase (GOX), resulting in AMPA, which is further degraded into inorganic phosphate and methylamine, with glyoxylate being produced, and subsequently degraded within the glyoxylate cycle [20]. The sarcosine pathway initiates with the direct cleavage of the C-P bond by the action of enzyme C-P lyase, leading to sarcosine and inorganic phosphate production [21]. The sarcosine oxidase enzyme further breaks down sarcosine to glycine, with its final environmental destiny being the complete mineralization to carbon dioxide and ammonium.

### 3.2. From Fields to Bloodstreams: Toxicological Relevance of Glyphosate Exposure

Widespread and intensive glyphosate use has resulted in increased environmental contamination and higher residue levels in plants. Glyphosate and its primary breakdown product, AMPA, are widely abundant in the environment. They can be found in water sources and nontarget plants, such as food products, especially soy and corn [11,22]. The environmental persistence is highest in Europe, South America, and East/South Asia, correlating with the use of genetically modified soybeans and corn [23]. The typical half-life of glyphosate in water ranges from a few days up to 91 days, while in various soil types it can extend up to 197 days. AMPA exhibits even greater environmental persistence, with a half-life ranging from 76 to 240 days [24]. Consequently, humans and other animals are continuously exposed through various routes that promote the adsorption by dermal contact, inhalation, and ingestion [25]. The average levels of glyphosate in human urine range from 0.26 to 73.5 µg/L in individuals with agriculture-related jobs and 0.16 to 7.6 µg/L in the general population, suggesting glyphosate is reaching domestic habitats [26]. An epidemiological study demonstrated the presence of glyphosate in the urine of the U.S. population, with detectable levels found in approximately 82% of individuals tested. The authors associated glyphosate exposure with endocrine disruption, as evidenced by inverse correlations between urinary glyphosate levels and blood concentrations of key sex hormones in both males and females [27]. In a biomonitoring pilot study conducted in Ireland, glyphosate was detected in 20% of urine samples collected from non-occupationally exposed adults, with concentrations ranging from 0.80 to 1.35 µg/L—values that exceeded those previously reported in several European studies, raising further concerns about environmental exposure in the general population [28]. Another retrospective biomonitoring study using 24-h urine samples from young German adults revealed that glyphosate was quantifiable in up to 57.5% of samples by 2012–2013, reflecting increasing environmental exposure trends over time, with a modest decline observed after 2014 [29].

Glyphosate exhibits a relatively short half-life in urine following oral exposure [30], which complicates its detection in single-time-point samples. Corroborating this study, the toxicokinetic profile of glyphosate showed a low urinary excretion rate, with only 1% of the dose recovered and a rapid elimination half-life of about 9 h. These findings also highlight the challenges of estimating dietary intake through urinary biomonitoring, given the limited excretion and swift clearance of glyphosate [31]. Nonetheless, the high frequency of positive results in random urine and blood samples in different studies suggests that exposure is likely continuous, highlighting the challenge of accurately assessing exposure levels. Therefore, the presence of glyphosate in biological samples has led to rising concerns and debates about adverse health outcomes across the population. Yet, a general discordance persists regarding the effects of glyphosate and GBHs on human health.

### 3.3. Controversies, Risk Assessments, and Potential Health Impact of Glyphosate

Over the past years, glyphosate has been the subject of regular assessments by various regulatory authorities, such as the European Food Safety Authority (EFSA) and, the U.S. Environmental Protection Agency (EPA), which have suggested that glyphosate and its primary breakdown product, AMPA, are unlikely to pose significant risk to mammals, falling under category IV, being practically non-toxic and non-irritating. This classification is based on the mechanism of action, which is restricted to plants and soil microorganisms, low environmental persistence, and toxicity data [16]. However, in 2015, the International Agency for Research on Cancer (IARC) classified glyphosate as a “probable human carcinogen”, based on epidemiologic evidence in humans, mainly for non-Hodgkin lymphoma [32]. Divergent conclusions among agencies, such as IARC, EPA, and EFSA, may stem from differences in methodological approaches, including the selection of studies (e.g., industry-sponsored vs. independent), criteria for data inclusion, and weight-of-evidence frameworks, which highlight the complexity and politicization of chemical risk assessments. In January 2018, the IARC published a formal response addressing the critiques received and reaffirmed its evaluation of glyphosate’s carcinogenic potential [33]. The contrasting interpretations and regulatory stances continue to fuel debate and uncertainty regarding the safety of glyphosate and its possible toxic and carcinogenic effects on human health.

The IARC also discovered compelling evidence indicating that glyphosate possesses two major traits associated with established human carcinogens: the induction of oxidative stress in cells and genotoxicity [34]. These disparities have led to general controversy and different regulatory strategies worldwide, ranging from complete prohibition to unrestricted policies over glyphosate use [35]. After the IARC publication, numerous risk assessment studies were carried out. The results suggested that glyphosate exposure can disturb the redox balance, which leads to loss of mitochondrial inner membrane potential and the activation of pathways associated with cell death. Ultimately, this may lead to the development of neurological disorders, such as autism, depression, Parkinson’s, and Alzheimer’s disease [36,37,38,39]. Moreover, glyphosate can negatively affect fertility by altering hormonal release and compromising the integrity of reproductive organs [40,41]. It is important to note that many studies reporting glyphosate-induced toxicity focus on GBH, which contains glyphosate as the active ingredient. These formulations also include other chemical components, such as surfactants, stabilizers, and diluents, designed to improve glyphosate’s efficacy by enhancing its absorption into the cells of target organisms. Therefore, the toxic effects of commercial formulations can also be associated with the adjuvants present in these products, which not only possess their own inherent toxicity but can also amplify the toxic effects of glyphosate [42]. Moreover, growing evidence highlights the toxicity of AMPA, the primary degradation product of glyphosate, which may also accumulate in water, soil, food products, and even human tissues [22,43,44]. Glyphosate, AMPA, and the adjuvants present in GBHs have been demonstrated to induce toxicological effects on non-target organisms and ecosystems [41,44,45,46]. Considering the potential interactions and synergistic effects between glyphosate, AMPA, and formulation adjuvants, it is also relevant to evaluate their toxicity separately, as well as the associated environmental and public health risks from pesticide exposure.

## 4. The Male Reproductive System as a Target of Glyphosate

**Figure 2:**
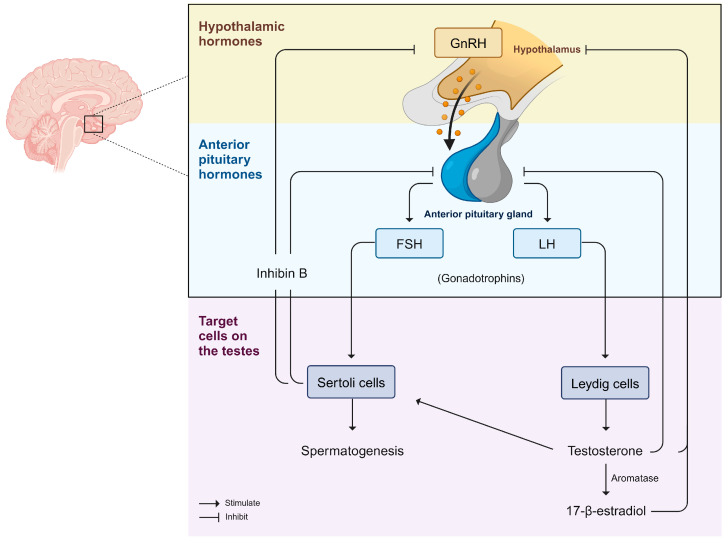
Schematic illustration of the hormonal regulation in the male reproductive system. Pulsatile secretion of gonadotropin-releasing hormone (GnRH) from the hypothalamus triggers the release of follicle-stimulating hormone (FSH) and luteinizing hormone (LH) from the anterior pituitary gland, which are responsible for the connection between the brain and the testis. FSH and LH stimulate Sertoli cells (SC) and Leydig cells (LC), respectively, leading to spermatogenesis and testosterone production. The production of inhibin B by SC and testosterone by LC provides a negative feedback loop that results in the reduction of GnRH, FSH, and LH production. All these factors and hormones play an important role in regulating spermatogenesis.

The male reproductive system provides the appropriate conditions for the development of germ cells, which are essential for the perpetuation of the species by the transmission of genetic as well as epigenetic information to successive generations [47]. Male reproductive function and health are mainly dependent on the testes, which perform essentially two functions: sex steroid hormone biosynthesis (steroidogenesis) and production of sperm (spermatogenesis). The testicular parenchyma is separated into an avascular spermatogenic compartment, the seminiferous tubules, recognized as the functional units of testes, and a highly vascularized endocrine compartment, the interstitial tissue, which contains Leydig cells (LC) [48]. The seminiferous tubules are compartmentalized by a complex junctional system between adjacent Sertoli cells (SC), creating one of the tightest blood-tissue barriers known to exist in mammalian tissues, the blood–testis barrier (BTB) [49]. This structure is responsible for controlling the movement of substances from the basal to adluminal compartment and acts as an immunological barrier by regulating the movement of immune cells and the level of cytokines in the seminiferous epithelium; it also acts as a physiological barrier composed of membrane transporters and ion channels. These components create a milieu responsible for the correct development of germ cells into fully developed spermatozoa [50]. Thus, the effect of diseases and environmental disruptors, such as glyphosate, on the metabolic co-operation between testicular cells may compromise spermatogenesis, and, consequently, male fertility potential. Testicular function is regulated by different hormones acting through both endocrine and paracrine pathways. These hormones regulate not only germ cell differentiation, but also the proliferation and function of the somatic cells necessary for the correct development of the testes [51]. In humans and other higher vertebrates, normal testicular function is highly dependent on coordinated endocrine action of the hypothalamic–pituitary–gonadal (HPG) axis (Figure 2) [52]. In brief, the hypothalamus secretes gonadotropin-releasing hormone (GnRH) in a pulsatile manner, stimulating the release of two hormones referred to as gonadotrophins: luteinizing hormone (LH) and follicle-stimulating hormone (FSH) from the anterior pituitary, which establishes the connection between the brain and the testes [53]. Gonadotrophins are then secreted into the systemic circulation and have distinct functions on the testes. LH stimulates the production of testosterone by LCs located in the interstitium, and FSH acts specifically on the SC, regulating the seminiferous tubules and stimulating spermatogenesis. Testosterone is also converted to 17-β-estradiol (E_2_) as a result of aromatase functions, and both of these hormones have a negative feedback effect on the hypothalamus and pituitary [54,55]. SC also produces some important sex steroids, inhibin B (inhibitory), activin (stimulatory), and other peptides, which, along with testosterone, regulate the HPG axis through feedback loops that limit GnRH and gonadotrophins production to maintain balance [56]. Normal spermatogenesis relies on the coordinated interaction between all these factors and hormones. Therefore, imbalances in the components of the HPG axis may have a significant impact on male reproductive health [57]. Perinatal exposure to glyphosate was demonstrated to increase gonadal activity and induce early onset of puberty in male offspring, which were associated with increased levels of testosterone, estradiol, LH, and FSH, alongside increased sperm. Such findings suggest that glyphosate may interfere with the endocrine and paracrine regulation of endocrine testicular function, with potential consequences for spermatogenesis [58].

**Figure 3:**
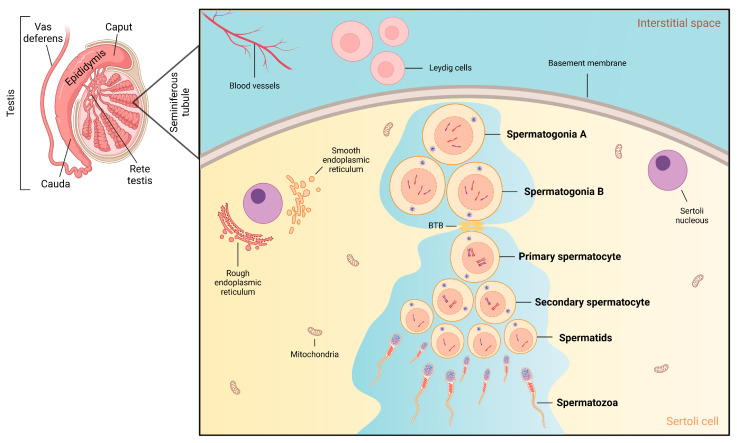
Schematic illustration of the principal events during spermatogenesis in male humans. Leydig cells and blood vessels are located in the interstitial space. The seminiferous epithelium is composed of Sertoli cells and developing germ cells. In the basal compartment, spermatogonia A undergo mitosis, developing into spermatogonia B, which enter the meiotic phase and differentiate into primary spermatocytes. In the apical compartment, primary spermatocytes undergo a first meiotic event, originating secondary spermatocytes. These secondary spermatocytes undergo a second meiotic event, where spermatids are formed. Spermatids migrate toward the lumen, where fully formed spermatozoa are finally released. Abbreviation: BTB—blood–testis barrier.

Spermatogenesis is a continuous physiological process that begins at puberty. It takes place in the epithelium of the seminiferous tubule, where type A spermatogonia include a subset of spermatogonial stem cells (SSCs) responsible for initiating and sustaining the process that ultimately leads to the formation of mature spermatozoa (Figure 3) [59]. SSCs are located in the basal compartment of the seminiferous epithelium and are defined like all other stem cells by the ability to produce more stem cells (self-renewal), to maintain the pool of stem cells through male life span, and by the ability to differentiate offspring (differentiation) [60]. To maintain a normal spermatogenesis, a balance between SSC self-renewal and differentiation is required. This balance is regulated by the surrounding microenvironment of stem cells, called the niche of the testis [61]. The niche milieu is provided by the somatic SC, the basement membrane, and cellular components of the interstitial space [62]. SC are the key element of the niche providing both nutritional and structural support for the development and maintenance of functional testis and the SSCs within them [63]. Exposure to environmental toxicants, such as glyphosate and glyphosate-based herbicides (GBH), has been demonstrated to disrupt spermatogenesis, leading to impaired sperm production and testicular alterations even at low doses [64]. Short-term exposure to high doses of GBH disrupts the SSC niche, leading to histopathological changes in the seminiferous epithelium and impairing spermatogenesis in adult Wistar rats, as evidenced by reduced androgen receptor (AR) expression, increased p53 activation, oxidative stress, and significant structural damage [65]. Additionally, glyphosate exposure has been shown to interfere with the meiotic processes. In vitro studies on pubertal male rats demonstrated that low concentrations of glyphosate disrupt the meiotic step, particularly affecting the formation and progression of spermatocytes [66].

## 5. Effects of Glyphosate on Male Reproductive System

Studies have indicated that glyphosate may have the potential to induce adverse effects in male reproductive health [41,64,65,66,67,68,69]. These effects have been demonstrated through in vivo, in vitro, and in silico experimental designs, demonstrating the pesticide’s potential to disrupt key reproductive processes. The in vivo studies, particularly those conducted on rodent models, have shown that glyphosate and GBHs can negatively impact endocrine and spermatogenic testicular function, leading to alterations in sperm quality, testosterone levels, and damage to the seminiferous epithelium. Some of the documented adverse effects of glyphosate on male reproductive health include decreased ejaculate volume, testosterone levels, testicular sperm production within the seminiferous epithelium, and sperm concentration and motility, and impaired sperm morphology due to an increase in the frequency of aberrant sperm cells [70]. In vitro studies have further elucidated the cellular and molecular mechanisms underlying these effects, highlighting the role of oxidative stress, mitochondrial dysfunction, and apoptosis in Sertoli and germ cells [41,67,71]. These findings suggest that glyphosate exposure can impair spermatogenesis and hormonal regulation, raising concerns about its potential risks to male fertility. The following sections will explore the documented reproductive health outcomes associated with glyphosate and GBHs exposure at different life stages in males, distinguishing between in vivo and in vitro evidence.

### 5.1. In Vitro Studies

In vitro studies on LC, SC, and germ cells have explored the mechanisms of glyphosate-induced toxicity in the testicular function, demonstrating disruptions in hormonal regulation, cell viability, and spermatogenesis (Table 1). Studies conducted in LC demonstrated decrease in testosterone production as well as the downregulation of steroidogenic genes and proteins, including Steroidogenic Acute Regulatory protein (StAR), Cytochrome P450 Family 17 Subfamily A Member 1 (CYP17A1), Cytochrome P450 Family 11 Subfamily A Member 1 (CYP11A1), and 3β-hydroxysteroid dehydrogenase, in response to exposure to glyphosate or GBHs [72,73,74]. A study conducted in the mouse LC line TM3 has shown that exposure to glyphosate at concentrations of 0.5 and 5 mg/L for periods ranging from 1 to 24 h inhibited testosterone secretion, with effects observed as early as 1 h post-exposure. At 5 mg/L, glyphosate also downregulated key enzymes involved in testosterone synthesis, such as StAR and CYP17A1, and induced endoplasmic reticulum stress via activation of the PERK/eIF2α signaling pathway [73]. Another study using the same cell lines demonstrated that 24 h exposure to 10 µM glyphosate (equivalent to 1.69 mg/L) inhibited testosterone synthesis by suppressing the expression of StAR and CYP11A1, thereby corroborating that glyphosate adversely affects male steroidogenesis. This study also showed that mitochondrial dysfunction, manifested by overproduction of mitochondrial reactive oxygen species (ROS), ultrastructural damage, and disturbance of mitochondrial dynamics, was responsible for the decreased protein levels of steroidogenic enzymes [72]. In the study by Zhao et al. [74], TM3 cells treated with glyphosate 0.1 mM (16.9 mg/L) revealed that the expression levels of StAR and CYP17A1 were significantly decreased, with reduced testosterone secretion. This study also showed that Nuclear Receptor Subfamily 1 Group D Member 1 (NR1D1) levels were upregulated, suggesting that glyphosate disturbs testosterone synthesis via NR1D1-mediated inhibition of StAR protein. Also, a study conducted in the MA-10 Leydig tumor cell line exposed to Roundup^®^ for 24 and 48 h observed the inhibition of steroidogenesis by the disruption of StAR protein expression [75]. Studies in placental cells have corroborated these findings, demonstrating that exposure to glyphosate formulations disrupted aromatase activity and mRNA levels by interacting with the enzyme’s active site. However, these effects were amplified by the commercial formulation Roundup^®^ in both microsomes and cell cultures [76], suggesting that the formulation adjuvants may enhance glyphosate’s bioavailability and/or bioaccumulation, which may lead to endocrine and toxic effects in mammals. Despite being derived from a non-testicular model, these findings corroborate the evidence for glyphosate’s classification as an endocrine-disrupting chemical (EDC). Together with the findings from testicular cell models, these results suggest that glyphosate’s endocrine-disrupting potential extends across different steroidogenic tissues.

Although the experimental conditions varied, in vitro studies using different murine Leydig cell lines (e.g., TM3, MA-10) consistently demonstrate that glyphosate and its commercial formulations impair testosterone synthesis. The underlying mechanisms appear to be multifactorial, involving not only the downregulation of key steroidogenic proteins like StAR and CYP17A1 but also the induction of endoplasmic reticulum stress via the PERK/eIF2α pathway and modulation of nuclear receptors like NR1D1 [69,72,73,74]. This convergence of findings, even at low concentrations, reinforces glyphosate’s classification as an EDC capable of directly targeting testicular steroidogenesis.

The differential sensitivity of testicular cell types to glyphosate and its commercial formulation Roundup^®^ was investigated through in vitro experimental models. Results showed that Roundup^®^ induced necrosis and apoptosis in LC from concentrations as low as 1 ppm (1 mg/L), showing significant cell membrane degradation and reduction of caspases 3 and 7 activities. Both glyphosate and Roundup^®^ decreased testosterone production, reinforcing the endocrine disruption effect even at low environmental exposure levels. Moreover, SC were primarily affected by glyphosate alone, while germ cells, exposed to higher doses of glyphosate, increased apoptosis, particularly in co-cultures with SC [69]. Another study demonstrated that glyphosate exposure reduced cell volume, increased cell dissociation, suppressed proliferation, enhanced apoptosis, and decreased expression of androgen-binding protein (ABP) and vimentin mRNAs in mouse SC [80]. Glyphosate and Roundup^®^ were also implicated in compromising the integrity of the SC junctional barrier, particularly by disrupting tight junctions that are critical components of the BTB, as evidenced by the reduction of transepithelial electrical resistance of these cells. Corroborating this finding, the authors demonstrated a delocalization of claudin11 from the cell membrane to the cytoplasm, although the expression levels of claudin11, ZO1, and occludin were not affected by pesticide exposure [67]. These cellular-level findings provide a mechanistic framework for understanding the systemic effects observed in whole-organism studies.

It has also been demonstrated that GBH formulations, such as Roundup Bioforce^®^ and Glyphogan^®^ (both formulations contain 360 g/L of glyphosate), exhibit higher cytotoxicity in TM4 SC than glyphosate alone. After 24 h of exposure, glyphosate alone (10–10,000 mg/L) did not significantly affect TM4 cell viability, whereas Roundup Bioforce^®^ induced marked cytotoxicity starting at 0.1% (360 mg/L glyphosate equivalent) and Glyphogan^®^ from as low as 0.005% (18 mg/L glyphosate equivalent). Lipid droplet accumulation was observed at 0.25–0.5% Roundup (900–1800 mg/L glyphosate), while Glyphogan^®^ at 0.05% (180 mg/L glyphosate) led to complete cell death. These effects, including mitochondrial dysfunction, oxidative stress, and glutathione-S-transferase inhibition (GST), are primarily attributed to polyethoxylated alkylamines (POEAs) and other co-formulants, rather than glyphosate itself [71]. Lipid droplet accumulation was also associated with testicular aging and degenerative dysfunction of SC [81]. Therefore, the accumulation of lipid droplets in SC induced by GBH might adversely impact male fertility. In this context, the accumulation of lipid droplets in SC following GBH exposure parallels the degenerative phenotype recently described in late-onset hypogonadism (LOH), in which aging-associated lipid-hoarding SC exhibit impaired support of spermatogenesis and reduced testosterone synthesis [81]. These converging findings suggest that exposure to glyphosate formulations may accelerate testicular aging and contribute to endocrine dysfunction.

It has been demonstrated that intracellular Ca^2+^ overload, oxidative stress, and disrupted signaling pathways were involved in the mechanism of Roundup^®^-induced SC toxicity at 36 mg/L, highlighting these cells as one of the glyphosate targets on testicular tissue, potentially affecting spermatogenesis and male fertility [41]. The study proposed that glyphosate-induced calcium overload, mitochondrial dysfunction, and oxidative stress were accompanied by activation of protein kinase C (PKC), Phosphoinositide 3-kinase (PI3K), Extracellular signal-regulated kinase 1 and 2 (ERK1/2), and p38 mitogen-activated protein kinase (MAPK) signaling pathways after 30 min of pesticide exposure. Moreover, antioxidant co-treatment with Trolox or ascorbic acid prevented Ca^2+^ influx and cell death, underscoring the role of oxidative imbalance in Roundup^®^-induced cytotoxicity. Together, these findings reveal that glyphosate formulations compromise Sertoli cell function via oxidative and calcium-mediated pathways, highlighting potential mechanisms underlying its endocrine-disrupting effects on male reproductive health.

Additionally, to the toxic effects of glyphosate on SC and LC, compelling evidence highlights that GBHs may have negative effects on human spermatozoa. Direct treatment of sperm to 1 mg/L of Roundup^®^ resulted in decreased sperm progressive motility after 1 h of incubation. Additionally, mitochondrial dysfunction was also a result of this treatment [77]. The same group observed a significantly reduced sperm progressive motility with the glyphosate treatment during the same period [79]. These two complementary studies investigated the effects of glyphosate and its commercial formulation Roundup^®^ on human semen from volunteers living in agricultural areas of Greece. In the first study, exposure to Roundup^®^ (1 mg/L) for 1 h led to a significant decrease in progressive sperm motility and mitochondrial dysfunction [77]. The second study used a glyphosate concentration of 0.36 mg/L, equivalent to the glyphosate content in the previously tested Roundup^®^ dose, and also observed reduced sperm motility and increased DNA fragmentation [79]. The authors highlighted that Roundup^®^ induced a more pronounced decline in sperm motility than glyphosate alone, suggesting that formulation components may potentiate glyphosate toxicity. Moreover, a study conducted in human sperm exposed to glyphosate 0.1–1000 nM for 1 h demonstrated a concentration-dependent disruption in the mitochondrial respiration efficiency, with inhibitory effects observed from concentrations of 100 nM (equivalent to 0.0169 mg/L). While initial reductions in active oxygen consumption (V3: state 3 respiration) were detected as early as 1 nM (0.000169 mg/L), significant decreases in mitochondrial efficiency (RCR) emerged only from 100 nM (0.0169 mg/L). At the highest tested concentration, glyphosate caused approximately a 31% reduction in RCR, representing a substantial impairment. Thus, glyphosate’s impact on sperm mitochondrial function becomes progressively more severe with increasing exposure [78]. Taken together, these findings highlight the need to evaluate the impacts of glyphosate on testicular cells, which collectively suggest a complex and multifaceted disruption of male reproductive function, potentially leading to impaired fertility and compromised reproductive health.

Despite the valuable mechanistic insights provided by using in vitro models, these protocols have inherent limitations. Unlike whole-organism experimental designs, in vitro assays bypass key pharmacokinetic processes such as absorption, distribution, metabolism, and excretion (ADME), resulting in direct and prolonged exposure of cultured cells to test substances. This may lead to overestimation of cytotoxic or endocrine-disrupting effects. Additionally, many cell lines exhibit limited or no metabolic capacity, impairing the evaluation of toxicant bioactivation or detoxification pathways that may occur in vivo. Consequently, toxic effects observed following in vitro protocols sometimes may differ in magnitude or mechanism from those seen in animal models or humans. These methodological constraints, as recognized by the Organization for Economic Co-operation and Development (OECD) Test Guidelines, suggest complementing in vitro findings with in vivo studies or human-relevant models to enhance translational relevance and risk assessment [82].

### 5.2. In Vivo Evidence of Glyphosate-Induced Male Reproductive Toxicity

A growing body of in vivo studies across diverse animal models has consistently demonstrated the disruptive potential of glyphosate and its commercial formulations on male reproductive systems (Table 2). It is worth noting that the reporting of glyphosate exposure metrics varied across studies. Some of them provide only the glyphosate concentration in drinking water or diet, whereas others report the administered dose per body weight. Without data on individual fluid or food consumption, direct conversion between these metrics is not possible, which may complicate cross-study comparisons. These studies provide systemic evidence of reproductive harm, which is mechanistically explained by the cellular-level disruptions discussed in the following sections.

In rodent models, exposure to glyphosate-based herbicides (GBHs) has been linked to significant testicular and hormonal changes. An acute 8-day exposure of reproductively mature Sprague–Dawley rats to Roundup^®^ (0.5% in water) resulted in increased levels of aromatase mRNA and protein in the testis and an increase in abnormal sperm morphology [83]. A concurrent study conducted in BALB/c wild-type (WT) mice showed profound inhibition of StAR and CYP17A1 expression following the oral administration of glyphosate for 4 weeks [74]. A study carried out by Romano et al. [68] reported that prepubertal oral exposure of male Wistar rats to a glyphosate-based formulation (5–250 mg/kg/day, gavage) delayed puberty onset, reduced serum testosterone, and induced seminiferous tubule alterations, even at the lowest tested.

Another study evaluated pubertal toxicity of soy milk supplemented or not with glyphosate (50 and 100 mg/kg) during the prepubertal period in male rats. Endocrine disruption was observed through a decrease in testosterone levels, concomitant with reduced SC number and an increase in the percentage of degenerated SC and LC in animals treated with soy milk and soy milk supplemented with glyphosate in both doses. Also, a decrease in the number of spermatids, an increase in the epididymal tail mass, and a decrease in the diameter of the seminiferous tubules were observed in animals receiving soy milk with glyphosate in both doses. Only animals receiving soy milk with 100 mg/kg glyphosate showed an increase in abnormal sperm morphology and a decrease in round spermatids [84]. In a subchronic in vivo study, sexually mature male Sprague–Dawley rats were daily exposed to glyphosate by oral gavage at doses of 5, 50, or 500 mg/kg for 5 weeks. While no significant histological alterations or changes in serum hormone levels were observed, the highest dose group (500 mg/kg) showed a significant reduction in total sperm count and seminal vesicle weight, suggesting potential impacts on male reproductive parameters at elevated exposure levels [85].

A 4-month exposure study in Sprague–Dawley rats fed glyphosate at 10 and 250 mg/kg demonstrated a significant decline in sperm quality and quantity, which was associated with glyphosate residues detected in serum (0.035–0.146 µg/mL) and testes (0.002–0.016 µg/g). These observations were concomitant with BTB disruption and increased testicular oxidative stress. Glyphosate exposure further increased ROS levels while decreasing the expression of antioxidative enzymes. These findings indicate that prolonged glyphosate exposure compromises male reproductive health not only by reducing sperm quality and quantity, but also through mechanistic pathways involving elevated ROS production, NADPH oxidase 1 (NOX1) upregulation, and estrogen receptor alpha (ER-α) activation. Such alterations collectively impair BTB integrity and mitochondrial function, providing mechanistic insight into glyphosate-induced testicular toxicity. In this study, glyphosate was incorporated into chow at concentrations of 10 and 250 mg/kg feed, corresponding to estimated doses of 0, 2, and 50 mg/kg BW/day, respectively. Dose estimates were calculated from the average feed intake (20 g/day) and initial body weight (100 g) of the animals. The low dose (2 mg/kg bw/day) was of the same order of magnitude as the U.S. EPA acceptable daily intake (1.75 mg/kg bw/day), whereas the high dose (50 mg/kg bw/day) represented 1/20 of the NOAEL (1000 mg/kg bw/day) established in rats [86]. Supporting these observations, studies on glyphosate-based herbicides (GBH) have documented congruent histopathological and biochemical changes in rat testicular cells. For example, GBH exposure has been shown to induce nitric oxide production and redox imbalance, increase p53 expression in Leydig and peritubular myoid cells, and concurrently reduce androgen receptor (AR) expression in Sertoli cells [65]. This disruption of cellular function aligns with other findings that GBH exposure increases blood–testis barrier (BTB) permeability in juvenile rats [87]. Collectively, the evidence highlights that oxidative stress serves as a central mechanism of testicular toxicity induced by both glyphosate and its commercial formulations [41,65,86].

Reproductive impairments have also been reported in non-rodent models, suggesting a conserved susceptibility across vertebrates. In birds, drakes (*Anas platyrhynchos*) exposed to Roundup^®^ by oral gavage of 5 or 100 mg/kg/day, for 15 days showed morphological alterations in the seminiferous tubules, epididymal ducts, and proximal efferent [88]. Also, there was a dose-dependent decrease in serum concentrations of 17β-estradiol and testosterone, with changes in the expression of androgen receptors (AR) specific to the testis [88]. Sexually mature male roosters exposed to Roundup for 5 weeks showed a significant increase in glyphosate and AMPA concentrations in seminal plasma, which was associated with lower sperm motility and higher levels of testosterone and oestradiol. In this study, glyphosate was incorporated into the diet at a concentration of 1250 mg/kg feed, with feed intake restricted to 200 g/day. Based on the average body weight of the, the estimated daily intake was 46.8 mg/kg bw/day, corresponding to approximately 47% of the EFSA-reported NOAEL (100 mg/kg bw/day) for poultry [89]. Conversely, a long-term study in Japanese quails (*Coturnix japonica*) found that while Roundup Flex^®^ (12–20 mg/kg) decreased plasma testosterone levels, it failed to affect overall reproductive functions [90], suggesting potential species-specific resilience. These findings suggest that long-term exposure to GBHs may disrupt endocrine function by reducing testosterone levels and altering gut microbiota composition, particularly during early developmental stages, without impairing reproductive output. Nonetheless, the observed hormonal and microbial shifts may have subtle, cumulative consequences on reproductive physiology. These outcomes highlight the relevance of such studies for predicting potential GBH-related risks to wildlife populations and the poultry industry [90]. In this study, glyphosate was administered via feed incorporation of a commercial GBH (Roundup Flex^®^) containing surfactants, rather than as pure glyphosate, to better mimic environmentally relevant exposures. The reported dose (12–20 mg/kg BW/day) represents an estimate based on the intended feed concentration (160 mg/kg feed) and typical intake rates in full-grown Japanese quails. Consequently, the individual glyphosate exposure could vary with feed consumption, and the combined presence of glyphosate and adjuvants precludes attribution of effects to the active ingredient alone.

**Table 2:** Summary of experimental conditions and main findings from studies investigating the in vivo effects of glyphosate in the male reproductive system.

Study Model	Compound	Dose	Period	Administration Route/Administered Medium	Main Results	Ref.
Mature male Sprague–Dawley rats	Roundup Grand Travaux Plus^®^	0.5% in drinking water (5000 mg/L) #	8 days	Oral ingestion; Diluted in a deionized water suspension	Increased levels of aromatase mRNA; Increased percentage of sperm with abnormal morphology.	[83]
Adult male Sprague–Dawley rats	Glyphosate	5, 50, 500 mg/Kg/day *	5 weeks	Oral gavage; Diluted in deionized water	At 500 mg/kg: ↓ total sperm count; ↓ seminal vesicle weight; No histological changes or significant effects on testosterone, estradiol, progesterone, oxidative stress markers (SOD, CAT, as well as MDA and H_2_O_2_ levels).	[85]
Sprague–Dawley rats (sperm from F3 generation)	Glyphosate	25 mg/Kg/day *	Gestational F0 exposure (From days 8 through 14 of gestation)	i.p. injection; Dissolved in PBS or DMSO	Sperm were collected from the lineage F3 generation males for epigenetic analysis; Identification of disease-specific differential DNA methylation regions (DMRs) and differential histone retention sites (DHRs) in sperm.	[91]
BALB/c wild-type mice	Glyphosate	0.5% (water) (5000 mg/L) #	4 weeks	Oral ingestion; Diluted in drinking water	Inhibition of StAR and CYP17A1 expression.	[74]
Prepubertal male Wistar rats	Roundup^®^	5, 50 or 250 mg/Kg/day *	30 days	Oral gavage; Diluted in water and administered once a day in a volume of 0.25 mL/100g BW	Morphological alterations in the testis; Delayed puberty onset; Decreased serum testosterone concentrations.	[68]
Adult drakes (*Anas platyrhynchos*)	Roundup^®^	5 or 100 mg/kg/day *	15 days	Oral gavage; Diluted in distilled water	Morphological alterations in the seminiferous tubules, epididymal ducts, and proximal efferent; Decreased serum concentration of 17β-estradiol and testosterone; Alterations in AR expression.	[88]
Male lizards (*Podarcis siculus*)	Glyphosate	0.05 and 0.5 µg/kg body weight	3 weeks	Oral gavage; Diluted in tap water and administered once a day 50 µL in via oral administration	Alterations in seminiferous tubules morphology; Alterations in estrogen receptors expression.	[92]
Adult male zebrafish (*Danio renio*)	Glyphosate	5 and 10 mg/L	24–48 h	Diluted in water	Decreased sperm motility; Decreased membrane and DNA integrity; Decreased mitochondrial function at the highest concentration.	[93]
Adult male zebrafish (*Danio rerio*)	Glyphosate	Estimated dose: 0.5, 5, and 50 mg/kg BW/day	21 days	Oral (dietary exposure)	At 0.5 mg/kg BW/day (EFSA ADI): impaired germ cell differentiation; histone acetylation changes. At 50 mg/kg BW/day (EFSA NOAEL): impaired steroidogenesis, DNA damage, reduced fertility.	[94]
Adult killifish (*Jenynsia multidentata*)	Roundup Original^®^ Roundup Transorb^®^ Roundup WG^®^	0.5, 1, or 5 mg/L of glyphosate	24–96 h	Diluted in water	Roundup Original^®^ increased ROS production; Decreased sperm motility at all studied concentrations.	[95]
Male Japanese quails (*Coturnix japonica*)	Roundup Flex^®^	Estimated dose: 12–20 mg/kg/day	50 weeks	Oral ingestion; Added in the organic feed at 160 mg/kg feed	Decreased plasma testosterone levels.	[90]
Ross 308 male roosters	Roundup^®^	Estimated dose: 46.8 mg/Kg/BW/ day	5 weeks	Oral ingestion; Added in fed: glyphosate 1250 mg/kg; AMPA 0.30 mg/kg	Increased levels of glyphosate and AMPA in seminal plasma compared to blood plasma; Decreased motility in spermatozoa; Increased testosterone and oestradiol levels.	[89]
Pregnant outbred Swiss mouse	Roundup 3 Plus^®^	0.5% (water) (5000 mg/L) Estimated doses: 0.5, 5, and 50 mg/kg/day	From E10.5 to 20 dpp	Diluted in drinking water	Alteration of testis morphology in 20 days old offspring; Decreased testosterone serum concentrations in 35 days old offspring and 8 months old mice.	[64]
Female Wistar rats	Glyphosate	50, 150 or 450 mg/kg	During pregnancy (21–23 days) and lactation (21 days)	Oral gavage; Diluted in distilled water and administered at 10 mL/Kg BW	Adverse reproductive effects on male offspring, such as decreased sperm counts; increased sperm abnormalities; decreased serum testosterone levels; signs of spermatid degeneration.	[96]
Female C57Bl/6 mice	Roundup Original DI^®^	0.5% (5000 mg/L); Estimated dose: 420 mg/kg BW/day	From the GD4 to the end of lactation period	Diluted in drinking water	Male offspring presented delayed testicular descent; Decreased number of spermatozoa.	[97]
4-week-old Sprague–Dawley male rats	Glyphosate	Estimated doses: 0, 2, and 50 mg/kg BW/day	4 months	Administered in chow (0, 10, and 250 mg glyphosate/kg chow)	Decreased sperm quality and quantity; Disrupted BTB integrity; Induced testicular oxidative stress; Increased ROS levels; NOX1 upregulation; ER-α activation.	[86]

The table includes only the most relevant studies for the scope of this review. The authors have selected and summarized only the most relevant findings from each study, based on their scientific judgment and the scope of the review. Abbreviations used: StAR—Steroidogenic Acute Regulatory protein; CYP17A1—Cytochrome P450 family 17 subfamily A member 1; BTB—Blood–testis barrier; ROS—reactive oxygen species; AMPA—Aminomethylphosphonic acid; GD4—Gestational day 4; NOX1—NADPH oxidase 1; ER-α—estrogen receptor alpha; DMSO—dimethyl sulfoxide (DMSO); PBS—phosphate buffered saline; ROS—reactive oxygen species; Ref: reference. * The administered volume or concentration of glyphosate in the exposure medium was not specified, limiting direct comparison with studies reporting exact exposure levels. Data were not available in the manuscript. # Data refer to glyphosate concentration in drinking water; actual dose (mg/kg/day) is unknown due to unreported fluid intake.

In aquatic and reptile models, similar toxic effects were observed. Male lizards (*Podarcis siculus*) exposed orally to glyphosate at 0.05 and 0.5 ug/kg body weight for 3 weeks showed alterations in the morphology of the seminiferous tubules, due to disruption of gap junctions, as well as in changes in the testicular expression of estrogen receptors, with these effects being mostly dose-dependent [92]. In reproductively mature zebrafish *Danio rerio* exposed to glyphosate diluted in water (5 and 10 mg/L), a decrease in sperm motility and motility period was observed. In this study, treatment with the highest concentration also resulted in reduced membrane and DNA integrity and mitochondrial functionality [93]. Additionally, a study carried out on male *Jenynsia multidentata* evaluated the effects of different variations of Roundup^®^, showing that sperm concentration and motility were affected by all herbicide treatments at a concentration of 0.5 mg/L of glyphosate [95]. Importantly, recent work using *Danio rerio* showed that dietary glyphosate exposure at doses within regulatory limits (0.5 mg/Kg/day; the acceptable daily intake, ADI) was sufficient to impair germ cell differentiation and induce histone acetylation changes in zebrafish testis [94], corroborating the relevance of non-monotonic dose-response patterns and highlighting the zebrafish as a sentinel species for assessing EDC-related effects in vertebrates. In the dietary exposure design of this study, the reported glyphosate doses (0.5, 5, and 50 mg/kg BW/day) represent estimated intakes based on feed incorporation and measured consumption rates, rather than direct administration of a fixed volume or concentration. Consequently, the exposure may vary among individuals depending on feed intake, and direct comparison with studies using waterborne administration should be interpreted with caution.

The impact of developmental exposure has also been a key area of investigation. Maternal exposure to glyphosate in rats and mice during pregnancy and lactation has been shown to cause adverse effects in male offspring, including delayed testicular descent, decreased sperm counts, increased sperm abnormalities, and decreased serum testosterone levels in puberty and adulthood. Pham et al. [64] carried out a study where pregnant mice were treated with glyphosate or Roundup^®^ at 0.5, 5, and 50 mg/kg/day. The results showed that exposure to this herbicide affects testis morphology in 20-day-old offspring and decreases serum testosterone concentrations in 35-day-old offspring and in 8-month-old mice. Moreover, a study conducted in female Wistar rats exposed to glyphosate during pregnancy and lactation (50, 150, or 450 mg/kg) showed adverse reproductive effects on male offspring, such as a decrease in sperm counts per epididymis tail and in daily sperm production during adulthood. Also, an increase in the percentage of abnormal sperm, a dose-related decrease in the serum testosterone level at puberty, and signs of individual spermatid degeneration were observed, suggesting that in utero and lactational exposure to glyphosate can induce adverse effects on the reproductive system of male Wistar rats at puberty and adulthood [96]. Additionally, Teleken et al. [97] conducted a study in female mice exposed to 0.5% Roundup in their drinking water from the fourth day of pregnancy until the end of the lactation period. Male offspring presented delayed testicular descent, decreased number of spermatozoa in the cauda epididymis, and reduced epithelial height of the seminiferous epithelium. Furthermore, maternal glyphosate exposure disrupts the HPG axis, enhancing LH secretion and increasing intratesticular testosterone levels, suggesting that glyphosate is an endocrine disruptor that may increase the risk of male infertility. Maternal glyphosate exposure during gestation and lactation has also been shown to decrease melatonin levels in rodent offspring during adulthood [98], further supporting its classification as an endocrine-disrupting chemical. Given the relevance of melatonin in supporting male reproductive health, primarily through its actions on the HPG axis, as well as its direct regulatory effects on testosterone synthesis by LC, this reduction is significant. Melatonin enhances sperm quality by exerting anti-apoptotic and antioxidant effects, mitigating ROS overproduction, and reducing testicular damage under conditions like diabetes, hypoxia, and obesity [99]. Therefore, glyphosate-induced decreases in melatonin may compromise male reproductive function, particularly by weakening the antioxidant defenses that are critical for maintaining testicular health and supporting spermatogenesis under oxidative stress conditions. Moreover, epigenetic alterations resulting from ancestral glyphosate exposure have been demonstrated in rodent models, with transgenerational changes in sperm DNA methylation and histone retention observed in the F3 generation, which were associated with an increased susceptibility to reproductive and metabolic disorders [91]. These findings highlight that perinatal exposure to glyphosate can malprogram the male reproductive system, with long-lasting consequences. Ancestral exposure to glyphosate can also cause transgenerational changes in sperm DNA methylation and histone retention in the subsequent generations of rats, which were associated with an increased susceptibility to disease.

### 5.3. Mechanistic Convergence of In Vivo and In Vitro Evidence

Data from systemic in vivo models indicate that glyphosate exposure is associated with reproductive toxicity. These findings are consistent with in vitro studies showing glyphosate induces cellular and molecular perturbations. Together, these lines of evidence provide a potential mechanistic basis for the observed testicular dysfunction, involving hormonal imbalances and compromised spermatogenesis.

Mechanistically, the downregulation of key steroidogenic genes, decreased testosterone and estradiol levels, and impaired spermatogenesis were consistent across most mammalian studies [66,72,82,90,91,92]. Some reports also indicated disruption of the BTB [93], altered expression of androgen and estrogen receptors [83,85], and increased oxidative stress and ROS production [87,93], suggesting overlapping yet diverse mechanisms of toxicity. While some mechanisms are well-supported, endpoints such as Ca^2+^ overload, mitochondrial dysfunction, and ferroptosis remain underexplored in vivo in reproductive tissue, despite their identification in in vitro models. Interestingly, GBH-induced intracellular Ca^2+^ overload has also been demonstrated in the hippocampus [100,101] and liver [102] following perinatal exposure. In the hippocampus, the involvement of NMDA receptor activation in mediating Ca^2+^ overload was identified [100], while in the liver, Ca^2+^ dysregulation was associated with intracellular iron accumulation, as well as increased iron levels in blood and bone marrow of offspring exposed to GBH during gestation and lactation, which were accompanied by inflammation [102]. Corroborating glyphosate’s endocrine-disrupting potential, perinatal exposure led to long-term reductions in circulating melatonin levels in adult offspring [98]. Although translational extrapolation remains challenging, these findings suggest that Ca^2+^ overload, oxidative stress, endocrine imbalance, and inflammation may converge to disrupt reproductive function. Considering that Sertoli cells express glutamate receptors and transporters, it is critical to investigate whether glyphosate-induced testicular toxicity involves glutamatergic signaling, particularly NMDA receptor overactivation. This pathway remains underexplored in the context of male reproductive health and may represent a novel target for future investigation. Supporting this hypothesis, a previous study has demonstrated that NMDA receptors are functionally expressed in testicular cells and participate in testosterone regulation via ERK signaling activation [103], suggesting that glutamatergic pathways may play a broader role in testicular endocrine homeostasis and could be disrupted by xenobiotics such as glyphosate.

Despite compelling preclinical evidence, translating these findings to human health risk is complicated by several factors related to study design and biological variability. A cross-species analysis of in vivo models reveals a consistent pattern of glyphosate- and GBH-induced male reproductive toxicity, yet with some variations depending on species, developmental stage, dose, and exposure duration. Reproductive impairments have been reported not only in classical rodent models exposed to the pesticide, but also in birds (e.g., *Anas platyrhynchos*), reptiles (*Podarcis siculus*), and fish (*Danio rerio*, *Jenynsia multidentata*) [88,92,93,95], suggesting a conserved susceptibility across vertebrates. Species-specific factors, including differences in hepatic cytochrome P450 enzyme activity, renal clearance rates, and transport mechanisms, critically modulate glyphosate bioavailability and toxicokinetics. These variables contribute to the divergent reproductive outcomes observed across experimental models and should be carefully considered when extrapolating findings to human health. Rodents, birds, and ruminants, for example, exhibit substantial differences in testicular architecture, spermatogenic dynamics, endocrine regulation, and antioxidant defense systems. Species-specific variations in the number of SC, permeability of the BTB, and expression of xenobiotic transporters can significantly influence glyphosate accumulation and tissue susceptibility. Moreover, interspecies differences in hormonal control of spermatogenesis and sensitivity to oxidative stress may modulate the cellular and molecular consequences of glyphosate exposure.

Another critical aspect in the studies included in this review is the heterogeneity of exposure protocols, including differences in glyphosate purity (pure compound vs. commercial formulations), dose, duration (acute vs. chronic), and administration routes (gavage, drinking water, chow), which limits direct comparisons. Many studies utilize high doses that, while useful for identifying toxicity thresholds, may not represent common environmental exposures, whereas chronic low-dose models are more realistic but can produce subtle effects. It is important to emphasize that although many experimental studies utilize glyphosate at doses significantly exceeding those encountered in environmental exposures, these high-dose models are useful for identifying toxicity thresholds, delineating molecular mechanisms (e.g., ferroptosis, mitochondrial dysfunction, calcium dysregulation), and evaluating potential protective interventions. Conversely, chronic low-dose exposure models more accurately reflect real-world human scenarios, especially occupational or dietary exposures, but often produce subtler physiological and biochemical changes. These effects may require sensitive biomarkers, extended observation periods, and refined experimental endpoints to be fully detected. Importantly, adverse effects reported at regulatory “safe” doses (ADI) [94] and evidence of non-monotonic dose-responses (a hallmark of EDCs) challenge the adequacy of current safety thresholds. Furthermore, the frequent use of high doses or artificial routes of administration (e.g., oral gavage or intraperitoneal injection) in experimental models—although valuable for elucidating mechanistic pathways such as ferroptosis, mitochondrial dysfunction, and calcium dysregulation—may not faithfully represent environmentally relevant human exposures. These limitations complicate direct extrapolation to human health risk and underscore the need for mechanistic studies employing human-relevant models, such as testicular organoids, ex vivo explants, and in silico toxicokinetic simulations, to bridge the translational gap.

Hence, despite preclinical evidence, translation of the studies to human health remains a challenge due to the complexity of human exposures, often involving mixtures of chemicals, chronic low-dose exposures, and interindividual variability. Growing evidence suggests that chronic exposure to glyphosate and its commercial formulations may contribute to male reproductive dysfunction by disrupting hormonal regulation, inducing oxidative stress, and promoting cellular damage in the testes (See Table 1 and Table 2). These effects can interact in a self-perpetuating cycle, wherein endocrine disruption and redox imbalance exacerbate testicular aging, metabolic dysfunction, and impaired spermatogenesis. Although in vitro and in vivo models have been instrumental in elucidating key mechanisms—such as reduced steroidogenesis, mitochondrial dysfunction, and blood–testis barrier disruption—translating these findings to humans remains challenging due to differences in exposure levels, species-specific physiology, and the complexity of human environmental co-exposures. Moreover, while antioxidant strategies show promise in mitigating these effects, further research is needed to define their efficacy, optimal targets, and translational relevance in real-world exposure scenarios. Studies should prioritize mechanistic insights, especially those involving oxidative stress—Ca^2+^ crosstalk, glutamatergic system, and ferroptosis, as well as standardized exposure designs across species to improve comparability and risk assessment relevance.

A key limitation of this review is the heterogeneity in the way glyphosate exposure is reported across studies. In some cases, only the administered dose per body weight (mg/kg/day) is provided, without the corresponding concentration in the exposure medium (e.g., mg/L in drinking water or diet). In other cases, only the concentration of glyphosate in the exposure medium is reported, without information on the actual administered dose, which depends on the amount of water or food consumed by the animals. In such cases, the dose was not included in this review because it was not reported in the original publications. This variability limits direct comparability between protocols using different routes or exposure matrices. Furthermore, values of glyphosate measured in biological fluids or tissues represent internal exposure and are not directly comparable to either administered dose or exposure concentration, as they reflect absorption, distribution, metabolism, and excretion processes. These differences should be taken into account when interpreting and comparing the results of different studies.

### 5.4. Glyphosate and Male Reproductive Toxicity: Mechanistic Insights with Emphasis on Oxidative Stress

**Figure 4:**
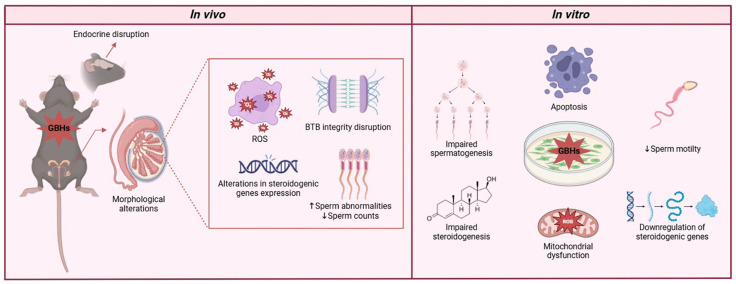
Overview of the effects of glyphosate and glyphosate-based herbicides (GBHs) on the male reproductive system, based on in vivo and in vitro studies. In vivo evidence suggests that glyphosate acts as an endocrine disruptor, impairing endocrine and spermatogenic testicular function through increased production of reactive oxygen species (ROS), dysregulation of steroidogenic gene expression, disruption of blood–testis barrier (BTB) integrity, and compromised sperm quality and quantity. Complementary in vitro studies have elucidated key molecular mechanisms, highlighting the involvement of oxidative stress, mitochondrial dysfunction, and apoptosis across various testicular cell types. Collectively, these findings support the potential of glyphosate and GBHs to interfere with critical reproductive processes.

Glyphosate has raised substantial concerns about its impact on human health, particularly about male reproductive toxicity. The herbicide’s environmental persistence and frequent detection in biological samples highlight the need to address its potential health risks. In this context, while exploring the mechanistic underpinnings of glyphosate’s toxicity is essential for addressing potential threats to male reproductive health, many of these mechanisms remain uncharacterized across the various cellular components of the male reproductive tract. Hence, we explored some of the key mechanisms through which glyphosate exposure may adversely affect male reproductive health, with a particular emphasis on its toxicokinetics, endocrine-disrupting properties, and impact on cellular functions in testicular tissues (Figure 4). Glyphosate’s impact on the male reproductive system is also mediated by its effects on Sertoli cells, which play a crucial role in supporting germ cell development and maintaining BTB. Glyphosate exposure has been shown to weaken the BTB by altering the expression and localization of tight junction proteins, thereby compromising the protective environment within the seminiferous tubules [67,86,87]. The damage to Sertoli cells and the BTB further highlights glyphosate’s potential to interfere with male fertility. Moreover, glyphosate exposure has been associated with apoptosis in LC, which are responsible for testosterone synthesis, further contributing to its endocrine-disrupting properties. The apoptosis of LC and subsequent hormonal imbalances can have profound effects on male reproductive health, potentially leading to decreased fertility and reproductive dysfunction.

One of the primary molecular mechanisms by which glyphosate induces these effects, impacting male reproductive health, is through oxidative stress. Oxidative stress, characterized by an imbalance between elevated levels of ROS and decreased activity of antioxidant mechanisms [41], seems to serve as a pivotal factor in GBHs’ toxicity. Glyphosate exposure has been shown to increase ROS levels in testicular cells, contributing to lipid peroxidation, protein oxidation, and DNA damage. Such oxidative stress can compromise sperm quality, reduce sperm motility, and increase apoptosis in Sertoli cells and LC. The role of oxidative stress in glyphosate-induced toxicity highlights the need for further research into potential protective strategies, such as antioxidants, which may mitigate these adverse effects.

In addition to oxidative stress, glyphosate exposure has been linked to genetic and epigenetic alterations, mitochondrial damage, and activation of inflammatory and cell death pathways, leading to neurological, metabolic, and reproductive diseases [36]. Mitochondria are critical for energy production and cellular homeostasis, particularly in energy-demanding processes such as spermatogenesis. Studies indicate that glyphosate exposure can impair mitochondrial function by disrupting the electron transport chain, leading to decreased ATP production and increased ROS generation. This mitochondrial impairment can result in reduced cell viability, altered metabolic activity, and increased apoptosis, all of which are detrimental to the health of testicular cells and the integrity of the male reproductive system.

Studies using animal models and cell lines demonstrated that dysregulation in Ca^2+^ homeostasis also plays a crucial role in GBH-induced cytotoxicity [41,104,105]. Mitochondrial Ca^2+^ homeostasis is critical for the regulation of mitochondrial metabolism, ATP production, and cell death [106]. This imbalance may arise from either the dysregulation of Ca^2+^ intracellular stores or from the increase in the membrane permeability to this ion. Depending on different tissues and cell types, GBHs can either increase or decrease intracellular Ca^2+^ levels, resulting in different outcomes. Higher Ca^2+^ concentrations induce cell death, while reduced intracellular Ca^2+^ levels activate proteins involved in cell proliferation and inhibit apoptosis. Previous studies have already shown an increase in intracellular Ca^2+^ levels in testes and SC [41], and also in other tissues or cellular systems, such as in the hippocampus [101], in rat liver [102], and in human renal cells [104]. Furthermore, an increase in ROS compromised antioxidant defenses, and oxidative damage to lipids and proteins was observed concurrently. Hence, intracellular Ca^2+^ overload is linked to oxidative stress caused by GBH exposure. Additionally, the activation of cell death pathways is marked by LDH release in the testes and hippocampus [41,101]. Moreover, exposure of the human renal proximal tubule cell line (HK-2) to glyphosate resulted in an increase in apoptotic cells. This increase was accompanied by the downregulation of the protein B cell lymphoma 2 (Bcl-2) and the upregulation of pro-apoptotic Bcl-2-associated X-protein (BAX), agonist of cell death (BAD), and caspase-3 [104]. Consequently, it appears that the increased influx of Ca^2+^ and oxidative stress in cells exposed to GBHs interact in a two-way mechanism, creating a feedback loop that exacerbates cellular damage, leading to the activation of cell death pathways.

GBHs increase intracellular Ca^2+^ concentration by activating voltage-dependent Ca^2+^ channels (L-VDCC) [104], N-methyl-D-aspartate (NMDA) receptors [100], and endoplasmic reticulum receptors, such as ryanodine. Given this, GBH-induced extracellular Ca^2+^ influx not only contributes to cytoplasmic Ca^2+^ overload but also elicits its release from the endoplasmic reticulum through the involvement of ryanodine receptors. The Ca^2+^ cytotoxicity induced by GBHs in rat testis also includes the activation of kinase cascades, such as PLC/PKC, PI3K, ERK1/2, and p38MAPK, which might be associated with the adaptive response to endoplasmic reticulum stress and/or ROS generation in the testis [41]. Corroborating the role of Ca^2+^ overload in glyphosate-induced cell toxicity, it has been demonstrated that increased Ca^2+^ influx, oxidative damage, and inflammation were associated with iron overload in liver, blood, and bone marrow of immature rats perinatally exposed to GBH, which may account for iron-driven hepatotoxicity [102]. Iron, as an essential transition metal in almost all living cells, plays a vital role in numerous biochemical processes. However, when iron accumulates within cells and tissues, it disrupts redox homeostasis, prompting the formation of ROS and leading to oxidative stress. Therefore, oxidative stress can cause tissue damage and contribute to the development of various diseases, with ferroptosis being a notable consequence at the cellular level [107]. Recent findings suggest that glyphosate may promote ferroptosis, an iron-dependent regulated cell death characterized by lipid peroxidation and glutathione (GSH) depletion [108]. This process is potentiated by mitochondrial dysfunction and impaired cystine uptake, which are hallmarks of oxidative damage. Additionally, disturbances in intracellular Ca^2+^ homeostasis induced by glyphosate may exacerbate mitochondrial permeability transition (MPT), ATP depletion, and ROS generation. Supporting this hypothesis, Bianco et al. [109] demonstrated that glyphosate exposure at 0.5 mg/L significantly decreased TMRE fluorescence in A172 glioblastoma cells, suggesting mitochondrial membrane potential collapse. Although this evidence was obtained in non-testicular tumor cells, it reinforces the plausibility of glyphosate-induced MPT and subsequent ROS generation. Liu et al. [108] demonstrated that glyphosate triggers hepatocyte ferroptosis via inhibition of the Nuclear factor erythroid 2–related factor 2 (Nrf2)/GSH/GPX4 axis, leading to GSH depletion and exacerbated liver injury, an effect reversible by N-acetylcysteine and ferroptosis inhibitors. Although conducted in hepatic models, these mechanistic insights reinforce the hypothesis that ferroptosis may also underlie testicular toxicity, especially in tissues with high oxidative metabolism. The integration of these pathways suggests a complex network of redox imbalance, excitotoxicity, and ferroptotic signaling contributing to male reproductive dysfunction following glyphosate exposure. Indeed, ROS generation can occur due to either physiological or pathological circumstances. Enzymatic and nonenzymatic antioxidants are crucial for preserving the redox balance and acting as a defense mechanism against ROS. Elevated ROS levels play a crucial role in the pathogenesis of numerous reproductive processes, given their potential harmful effects on sperm quality and function. Moreover, excessive ROS production can lead to DNA damage, hastening germ cell death and decreasing sperm counts, which may contribute to male impaired fertility [110,111].

Building on this, we propose a novel pathway where these mechanisms may be causally linked. We hypothesize that glyphosate-induced Ca^2+^ overload could trigger mitochondrial permeability transition and dysfunction, subsequently leading to increased ROS generation and the depletion of cellular antioxidants like glutathione (GSH). In an iron-rich cellular environment, this combination of depleted GSH and high levels of lipid ROS would establish the canonical conditions for ferroptosis by impairing the activity of the critical antioxidant enzyme GPX4. Therefore, we suggest that glyphosate-induced testicular cell death may occur via an integrated cascade: Glyphosate-induced Ca^2+^ overload → Mitochondrial dysfunction → ROS generation and GSH depletion → GPX4 inactivation and lipid peroxidation → Ferroptotic cell death. While this hypothesis integrates reported observations and provides a coherent framework for further investigation, we acknowledge that direct evidence of glyphosate-induced MPT is currently lacking and warrants experimental validation.

Still, while evidence connects glyphosate to reproductive harm, a regulatory consensus remains elusive. This divide stems, in part, from a lack of clear understanding regarding the precise molecular mechanisms underlying its effects. The disparity between studies reporting minimal risk and those highlighting cumulative low-dose impacts underscores the need for refined risk assessments and regulatory guidelines. These must prioritize elucidating glyphosate’s endocrine-disrupting potential and its specific molecular impact on reproductive health, bridging the existing mechanistic knowledge gaps.

### 5.5. Redox Imbalance and Antioxidant-Based Protection in Glyphosate-Induced Testicular Toxicity

The testicular tissue is particularly vulnerable to oxidative insults due to its high metabolic rate, extensive mitochondrial activity, and the abundance of polyunsaturated fatty acids in germ cell membranes. Exposure to glyphosate has been linked to increased production of ROS, enhanced lipid peroxidation, and suppression of key antioxidant enzymes such as glutathione peroxidase (GPx), catalase (CAT), and superoxide dismutase (SOD) in testicular tissue [41,86,100,111,112]. These oxidative disturbances disrupt the homeostasis of SC and LC, leading to impaired spermatogenesis and reduced sperm quality. Evidence suggests that glyphosate-induced oxidative stress may activate redox-sensitive intracellular signaling pathways, including the Nrf2/Keap1 axis [108,112,113] and MAPK cascades (such as p38, c-Jun N-terminal kinase-JNK, and ERK) [41,101], leading to impaired antioxidant defenses, inflammation, and apoptosis in different cell types, including testicular ones. These mechanisms have been observed in various experimental models of glyphosate-induced toxicity and are recognized as critical contributors to reproductive dysfunction. Accordingly, these pathways represent promising molecular targets for antioxidant-based therapeutic interventions.

In a recent study using breeder roosters, trehalose supplementation counteracted glyphosate-induced testicular damage by restoring redox homeostasis. Glyphosate exposure elevated ROS and lipid peroxidation, reduced antioxidant enzyme activities (CAT, SOD, GPx), and suppressed Nrf2 signaling in testicular tissue, while trehalose administration reversed these alterations, highlighting its potential as an antioxidant modulator of the Nrf2 pathway [112]. Additionally, N-acetylcysteine has been demonstrated as an effective antioxidant able to mitigate glyphosate-induced oxidative stress and tissue damage in multiple organs, including the testis, by restoring glutathione levels, reducing lipid peroxidation, preventing intracellular calcium overload, and improving histopathological outcomes [102,114,115]. The protective effects on spermatogenesis and male reproductive function highlight its potential as a therapeutic agent against oxidative insults from environmental toxicants [116]. In adult male albino rats, subchronic oral exposure to glyphosate (375 mg/kg/day) for 6 weeks led to reproductive toxicity, characterized by decreased sperm count, motility, and viability, alongside increased sperm abnormalities and testicular oxidative stress. Co-administration of N-acetylcysteine (160 mg/kg/day) significantly mitigated glyphosate-induced testicular damage by restoring antioxidant capacity, upregulating Nrf2 expression, reducing lipid peroxidation, and suppressing apoptotic markers (cytochrome c, Bax, and caspase-3), demonstrating its protective role through modulation of redox and apoptotic pathways [114]. Consistent with these findings, N-acetylcysteine also restored redox homeostasis and reduced glyphosate-induced cytotoxicity in vitro at 10 mM (1.69 g/L) in caprine testicular cells [117,118]. While these in vivo and in vitro studies collectively support the protective role of N-acetylcysteine against glyphosate-related reproductive damage, it is important to emphasize that direct dose-to-concentration extrapolations are not feasible in the absence of pharmacokinetic data. Accordingly, the comparisons presented here are qualitative, intended to highlight consistent mechanistic findings rather than to imply quantitative equivalence of exposure levels.

In rats, oral exposure to glyphosate (LD50/10, 787.85 mg/Kg body weight) induces pro-oxidant effects in testicular tissue, characterized by increased lipid peroxidation, elevated oxidant status, and depletion of key antioxidant defenses, such as glutathione, leading to structural and functional impairments in male reproductive cells. In contrast, hesperidin exhibits potent antioxidant activity, effectively restoring redox balance, reducing oxidative biomarkers, and reversing glyphosate-induced testicular damage, highlighting its potential as a protective agent against pesticide-induced reproductive toxicity [119]. Similarly, proanthocyanidin demonstrated antioxidant and cytoprotective properties under the same pesticide exposure conditions (LD_50_/10 of glyphosate), attenuating oxidative damage and preserving testicular function [120]. These findings further highlight the pharmacological relevance of natural polyphenolic compounds as potential therapeutic agents for mitigating glyphosate-induced reproductive toxicity.

Ascorbic acid (vitamin C, 200 mg/Kg) has also demonstrated protective effects against glyphosate-induced testicular toxicity (250 and 500 mL/Kg). Its antioxidant properties help restore redox balance by reducing lipid peroxidation and enhancing endogenous antioxidant defenses, thereby mitigating oxidative damage induced by glyphosate to germ cells and preserving sperm quality [121]. The protective role of antioxidants against glyphosate-induced toxicity is further supported by evidence showing that melatonin mitigates cognitive and affective impairments triggered by perinatal exposure to glyphosate-based herbicides (75 mg/Kg body weight). These effects are mediated through redox-modulating pathways, including the reduction of lipid peroxidation and the normalization of catalase activity in both male and female adult rats [120]. Vitamin C and vitamin E have been shown to counteract glyphosate-induced oxidative damage by reducing lipid peroxidation and preventing intracellular calcium overload and necrosis in Wistar rat testis and primary SC cultures, supporting their protective role against impaired redox homeostasis [41].

**Figure 5:**
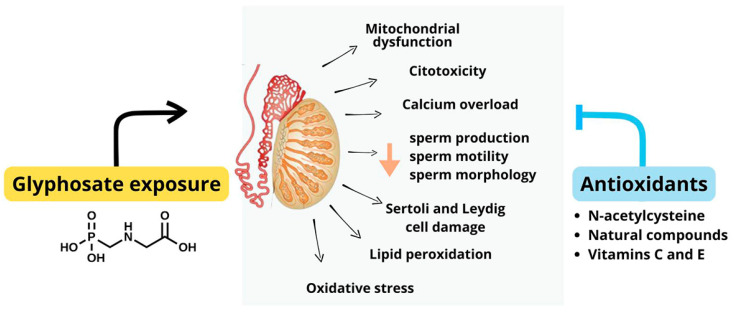
Schematic representation of glyphosate-induced testicular toxicity and the potential protective role of antioxidant-based interventions in mitigating the deleterious effects of the pesticide and preserving testicular structure and function.

Although the dose of glyphosate used in most of the studies exceeds typical environmental exposure levels observed in agricultural or residential settings, it remains relevant for understanding the mechanisms of acute toxicity. Such high-dose models are commonly employed to mimic conditions of accidental or occupational poisoning, enabling the identification of key biological targets, such as redox imbalance and testicular damage, and the evaluation of potential therapeutic strategies. In this context, the findings provide valuable insights into the pro-oxidant effects of glyphosate under toxicological stress and highlight the efficacy of antioxidants in mitigating herbicide-mediated reproductive toxicity (Figure 5).

## 6. Conclusions

**Figure 6:**
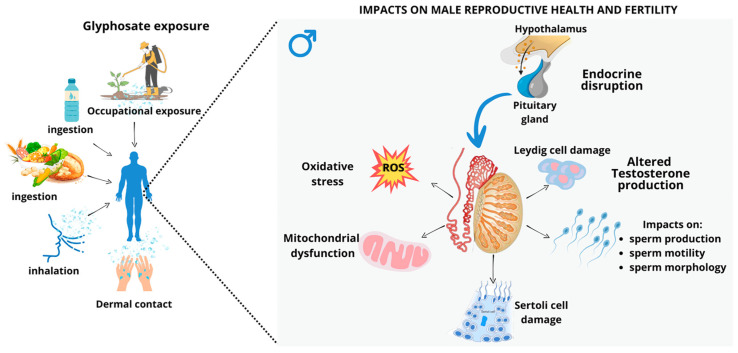
Representative illustration of the potential effects of glyphosate exposure on male reproductive health and fertility. Glyphosate exposure can occur through occupational activity, ingestion of contaminated water and/or food, inhalation, and dermal contact. Glyphosate exposure has been linked to disruptions in the endocrine system, particularly affecting the hypothalamic–pituitary–gonadal axis. This disruption might impair Leydig cell function, altering testosterone production. These hormonal imbalances may compromise spermatogenesis and negatively affect sperm parameters, including sperm count, motility, and morphology. In parallel, glyphosate-induced oxidative stress and mitochondrial dysfunction, along with damage to Sertoli and Leydig cells (SC and LC), further contribute to testicular toxicity. Although these effects have been primarily observed in experimental models, they raise concerns about potential risks to human male reproductive health and fertility.

The evidence presented in this review suggests that glyphosate exposure poses a significant risk to male reproductive health, with potential implications for fertility and hormonal balance (Figure 6). The herbicide’s ability to disrupt endocrine function, induce oxidative stress, impair mitochondrial function, and damage testicular cells highlights the complexity of its impact on the reproductive system. Given the widespread use of glyphosate and the prevalence of detectable levels in biological samples, there is a pressing need for further research to elucidate its long-term effects on human health.

Future studies should focus on examining the chronic effects of low-dose exposure, exploring potential protective interventions, and refining biomonitoring techniques, as these approaches would better mirror real-world human exposure scenarios and improve risk assessment in vulnerable populations. In this context, several antioxidant agents—including vitamin C, vitamin E, melatonin, hesperidin, proanthocyanidin, and N-acetylcysteine—have demonstrated protective effects against glyphosate-induced testicular damage in both in vitro and in vivo models. These compounds act by restoring redox balance, enhancing endogenous antioxidant defenses, reducing lipid peroxidation, and preserving germ cell structure and function. Such findings support the therapeutic relevance of antioxidants as complementary strategies to mitigate herbicide-mediated reproductive toxicity.

In summary, evidence implicates oxidative stress as a pivotal pathway in glyphosate-induced male reproductive toxicity. The disruption of redox homeostasis and antioxidant defense systems appears to underlie several downstream effects, including hormonal imbalances, mitochondrial dysfunction, and impaired spermatogenesis. These findings reinforce the relevance of redox biology and support the potential application of antioxidant strategies as protective agents against pesticide-induced reproductive harm.

Ultimately, addressing the reproductive health risks associated with glyphosate exposure requires a collaborative effort among researchers, public health officials, and regulatory agencies. By advancing our understanding of glyphosate’s toxicological profile and its impact on male reproductive health, we can better inform public health policies and safeguard human health in the face of ongoing environmental contamination.

## 7. Limitations

This review is subject to several limitations. The inclusion of studies with different experimental designs, species, exposure routes, and formulations of glyphosate introduces variability in the observed effects. Furthermore, although many studies utilize glyphosate doses that exceed environmentally relevant levels, such high-dose models remain valuable for elucidating toxicological mechanisms and for assessing the effects of acute or accidental intoxication, including the extent of tissue damage and the identification of potential therapeutic interventions. Additionally, although antioxidant strategies show promise in preclinical models, their efficacy, dosage, and safety in humans remain to be established through controlled clinical trials. Moreover, in some in vivo studies, only the administered dose per body weight (mg/kg/day) was reported, without the corresponding concentration in the exposure medium (mg/L in water or diet). This limits direct comparability between protocols using different routes or exposure matrices. Furthermore, internal concentration values (in biological fluids or tissues) might not be directly comparable to administered doses, as they reflect absorption, distribution, metabolism, and excretion processes. These differences should be considered when interpreting and comparing results. Lastly, the predominance of animal and in vitro studies highlights the need for epidemiological and biomonitoring data to better assess human reproductive risks associated with glyphosate exposure.

Despite these limitations, the collective findings reinforce the need for regulatory vigilance, public health awareness, and ongoing research to evaluate glyphosate’s reproductive toxicity and the potential role of antioxidant interventions in mitigating its adverse effects.

## Figures and Tables

**Table 1:** Summary of experimental conditions and main findings from studies investigating the in vitro effects of glyphosate on Leydig, Sertoli, and germ cells, highlighting the cellular and molecular mechanisms involved in pesticide-induced toxicity in the male reproductive system.

Study Model	Compound	Concentration Used	Period of Treatment	Main Results	Ref.
TM3 mouse Leydig cell line	Glyphosate	0.5 mg/L 5 mg/L	24 h	Inhibition of testosterone secretion; Downregulation of testosterone synthase StAR and CYP17A1; Endoplasmic reticulum stress; Activation of PERK/eIF2α signaling pathway.	[73]
TM3 mouse Leydig cell line	Glyphosate	10 µM (equivalent to 1.69 mg/L)	24 h	Inhibition of testosterone synthesis; Suppression of StAR and CYP11A1 expression levels; Overproduction of mitochondrial ROS; Ultrastructural damage; Disruption of mitochondrial dynamics.	[72]
TM3 mouse Leydig cell line	Glyphosate	0.1 mM (equivalent to 16.9 mg/L)	24 and 48 h	Decrease in StAR and CYP17A1 expression levels; Upregulation of NR1D1 levels.	[74]
MA-10 Leydig tumor cell line	Roundup^®^	25 µg/mL (equivalent to 25 mg/L)	2–4 h	Inhibition of steroidogenesis.	[75]
Sertoli cells isolated from 20-day-old Sprague–Dawley rats	Glyphosate Roundup^®^	10, 100, and 1000 ppm (equivalent to 10, 100, and 1000 mg/L)	48 h	Signal delocalization from membrane to cytoplasm.	[67]
Testis and Primary culture of Sertoli cells isolated from 30-day-old Wistar rats	Glyphosate Roundup^®^	0.036 g/L Roundup or glyphosate (equivalent to 36 mg/L)	30 min	Increased ^45^Ca^2+^ uptake; Necrosis; Oxidative stress: GSH depletion, lipid peroxidation, protein carbonylation, and enhanced activity of antioxidant enzymes (GPx, GR, GST, G6PD, γGT, CAT, SOD); Mechanism of toxicity dependent on PLC/PKC, PI3K, ERK1/2, and p38MAPK pathways; Effects of the pesticide were prevented by antioxidants Trolox and ascorbic acid.	[41]
Mature rat fresh testicular cells from Sprague–Dawley rats (Leydig, Sertoli, and germ cells)	Glyphosate Roundup^®^	1–10,000 ppm (equivalent to 1–10,000 mg/L)	1–48 h	Induced necrosis and apoptosis in LC; Reduced caspases 3 and 7 activity; Decreased testosterone production.	[69]
TM4 mouse Sertoli cell line	Glyphosate Roundup Bioforce^®^ Glyphogan	10 ppm–1000 ppm (equivalent to 10–1000 mg/L)	24 h	Reduced cell viability; Mitochondrial dysfunction; Oxidative stress; Lipid droplet accumulation.	[71]
Human semen from 66 volunteers living in an agricultural region in Greece	Roundup^®^	1 mg/L	1 h	Reduced sperm progressive motility; Mitochondrial dysfunction.	[77]
Human spermatozoa	Glyphosate	0.1–1000 nM (equivalent to 0.0000169–0.169 mg/L)	1 h	Disruption of mitochondrial respiration efficiency.	[78]
Human semen from 30 healthy volunteered men living in an agricultural region in Greece	Glyphosate	0.36 mg/L	1 h	Decreased progressive motility of sperm; Reduced motility; DNA fragmentation	[79]

The table includes only the most relevant studies for the scope of this review. The authors have selected and summarized only the most relevant findings from each study, based on their scientific judgment and the scope of the review. Abbreviations used: StAR—Steroidogenic Acute Regulatory protein; CYP17A1—Cytochrome P450 family 17 subfamily A member 1; CYP11A1—Cytochrome P450 family 11 subfamily A member 1; PERK—PKR-like Endoplasmic Reticulum Kinase; eIF2α—Eukaryotic Initiation Factor 2 alpha; NR1D1—Nuclear Receptor Subfamily 1 Group D Member 1; GSH—Reduced Glutathione; GPx—Glutathione Peroxidase; GR—Glutathione Reductase; GST—Glutathione S-Transferase; G6PD—Glucose-6-Phosphate Dehydrogenase; γGT—Gamma-Glutamyl Transferase; CAT—Catalase; SOD—Superoxide Dismutase; LC—Leydig Cells, Ref—references.

## Data Availability

No new data were created or analyzed in this study. Data sharing is not applicable to this article.

## Abbreviations

The following abbreviations are used in this manuscript:

ADI	Acceptable daily intake
ADME	Absorption, distribution, metabolism, and excretion
AMPA	Aminomethylphosphonic acid
BAD	Associated agonist of cell death
BAX	Pro-apoptotic Bcl-2 associated X-protein
Bcl-2	Protein B cell lymphoma 2
BTB	Blood–testis barrier
BW	Body weight
CAT	Catalase
CYP11A1	Cytochrome P450 family 11 subfamily A member 1
CYP17A1	Cytochrome P450 family 17 subfamily A member 1
DMSO	Dimethyl sulfoxide
EDC	Endocrine-disrupting chemical
EFSA	European Food Safety Authority
EPA	U.S. Environmental Protection Agency
eIF2α	Eukaryotic Initiation Factor 2 alpha
EPSPS	5-enolpyruvylshikimate-3-phosphate synthase
ER-α	Estrogen receptor alpha
FSH	Follicle-stimulating hormone
G6PD	Glucose-6-Phosphate Dehydrogenase
GBH	Glyphosate-based herbicide
γGT	Gamma-Glutamyl Transferase
GnRH	Gonadotropin-releasing hormone
GPx	Glutathione Peroxidase
GR	Glutathione Reductase
GSH	Reduced Glutathione
GST	Glutathione S-Transferase
HK-2	Human renal proximal tubule cell line
LC	Leydig cells
LH	Luteinizing hormone
LOH	Late-onset hypogonadism
MPT	Mitochondrial permeability transition
NMDA	N-methyl-D-aspartate
NOX1	NADPH oxidase 1
NR1D1	Nuclear Receptor Subfamily 1 Group D Member 1
Nrf2	Nuclear factor erythroid 2–related factor 2
OECD	Organization for Economic Co-operation and Development
PBS	Phosphate buffered saline
PERK	PKR-like Endoplasmic Reticulum Kinase
POEA	Polyethoxylated alkylamines
ROS	Reactive oxygen species
SC	Sertoli cells
SSCs	Spermatogonial stem cells
SOD	Superoxide Dismutase
StAR	Steroidogenic Acute Regulatory protein
VDCC	Voltage-dependent calcium channels
